# Exploring the Relationship Between Medicine Related Beliefs and Side‐Effect Experience Among White Oral Contraceptive Users in the UK

**DOI:** 10.1111/psrh.70012

**Published:** 2025-05-27

**Authors:** Lorna Reid, Rebecca K. Webster

**Affiliations:** ^1^ Department of Psychology University of Sheffield, ICOSS Sheffield UK

**Keywords:** discontinuation, medicine beliefs, nocebo effect, oral contraceptives, side effects

## Abstract

**Objectives:**

Side‐effects are often central to the decision to discontinue oral contraceptives. However, many oral contraceptive side‐effects may be the result of a psychological nocebo effect. In this preliminary study, we investigate whether correlates of nocebo effects are associated with oral contraceptive side‐effect experience.

**Design:**

An exploratory online cross‐sectional survey of 275 female, predominantly young, White respondents was conducted. Associations between psychological factors previously implicated in nocebo responses (beliefs about medicines, perceived sensitivity to medicines, side‐effect expectations, medicine information seeking, anxiety and trust in medicines), and oral contraceptive side‐effect experience were assessed using regression analyses.

**Results:**

Increased side‐effect expectations, stronger beliefs that medicines cause harm and are overused, increased perceived sensitivity to medicines, and decreased trust in medicine development were associated with increased attribution of symptoms to the oral contraceptive. Higher side‐effect attribution scores were also associated with discontinued oral contraceptive use.

**Conclusion:**

These preliminary findings demonstrate a potential role that nocebo‐related factors may have in impacting oral contraceptive side‐effect experience. Importantly, these factors are amenable to psychological interventions which could be employed to reduce oral contraceptive side‐effect experience and, as a result, unnecessary discontinuation. Future research must first assess such relationships using a prospective design to confirm the direction of the associations identified using more diverse samples of oral contraceptive users to increase the generalisability of findings.

## 1 Introduction

Despite being the leading form of contraception, as many as 60% of oral contraceptive users discontinue oral contraceptives within 24 months of initiation [1, 2, 3], often switching to a different method of contraception which can be less effective or abandoning contraceptive use entirely, increasing chances of pregnancy. Side‐effects are often central to poor adherence and discontinuation of oral contraceptives [4, 5].

However, many side‐effects reported by oral contraceptive users are non‐specific, such as acne, breast tenderness, nausea which may not be due to the pharmacological action of the oral contraceptive. Indeed, a review of seven randomized placebo‐controlled trials found there was no significant differences in reported side‐effects when participants were administered a placebo or an active oral contraceptive [6]. This demonstrates plausibility for oral contraceptive side‐effects to be psychological in origin rather than pharmacological, caused by a “nocebo effect.” The nocebo effect occurs when symptoms are attributed to an exposure but are not directly caused by the physical properties of the exposure itself [7]. This may facilitate the understanding of non‐specific side− effects which are attributed to a medication, when the effects experienced are not produced by the pharmacological action of the medication [8].

One of the main mechanisms driving these nocebo effects is negative expectations [9]. For example, prior expectations about side effects from medication can be self‐fulfilling and lead to the experience and attribution of side−effects to the medication. Research has suggested that these negative expectations can be exacerbated by a number of factors such as anxiety [10, 11], medicinal‐related beliefs [12, 13, 14, 15], and media influences which have been associated with nocebo effects to treatment [7, 16, 17, 18]; however, in the context of OC use specifically, this area is under‐researched.

To address the limited research in this area, we conducted an initial exploratory study to test whether these factors are also related to an individual's experience of oral contraceptive side‐effects and the effect of experienced side‐effects on discontinuation rates. This may provide an avenue for future research to conduct more thorough investigations of the influence these factors have on individuals' oral contraceptive experiences, with the potential for interventions to improve patients' oral contraceptive experiences and consequently reduce discontinuation and the risk of unintended pregnancies.

## 2 Methods

### 2.1 Respondents

Respondents participated through opportunity sampling via adverts on social media and the University Online Research Participation System between November 21–December 15, 2020. The adverts called for females to complete a survey about their experiences using oral contraceptives in the last 18 months. Respondents were eligible if they were female, fluent in English, and had taken the contraceptive pill in the last 18 months. Ethical approval was obtained from the University of Sheffield (withheld of for peer review) Ethics Board (Reference number 037165). All respondents provided electronic informed consent before starting the study.

An a priori calculation was calculated based on linear regression analyses to detect a small to medium effect size (*f* ^2^ = 0.11), based on a previous study investigating the effect of medication beliefs on side‐effect reporting in rheumatoid arthritis patients [14]. Using this estimate (assuming *α* = 0.05, power at 90%), a minimum sample size of 98 was required.

### 2.2 Design

The study used a cross‐sectional survey design to identify associations between psychological factors and oral contraceptive experiences in the general population.

### 2.3 Measures

All measures were collected using an anonymous survey on the platform Qualtrics [19].

#### 2.3.1 Predictors

To measure beliefs about medicines, we used the overuse and harm general subscales of the Beliefs about Medicines Questionnaire [20]. These included four items on medicines overuse (cronbach's alpha = 0.73), and four items on harm (cronbach's *α* = 0.58), rated on a 5‐point scale from “Strongly disagree” (1) to “Strongly agree” (5). Higher scores indicate greater beliefs that medicines are overused and cause harm.

Perceived sensitivity to medicine was measured using the Perceived Sensitivity to Medicines Questionnaire [21], consisting of five items (cronbach's *α* = 0.85), rated on a 5‐point scale from “Strongly disagree” (1) to “Strongly agree” (5). Higher scores indicate greater perceived sensitivity to medicine.

Medicine information seeking was assessed using two questions from prior studies: “How often do you read the information sheets in medicine packs?” and “How often do you look up medicine information on the internet?” [22, 23] (cronbach's *α* = 0.63), rated on an 11‐point scale from 0 (Never) to 10 (Always). Higher scores indicate greater information‐seeking.

Side‐effect expectations was assessed using the single‐item “When taking medication, I expect to experience associated side effects”, rated on a 5‐point scale from “Strongly disagree” (1) to “Strongly agree” (5). Higher scores indicate greater side‐effect expectations.

Anxiety was measuring using the Short‐form Spielberger State–Trait Anxiety Inventory [24] consisting of five items (cronbach's *α* = 0.85), rated on a 4‐point scale ranging from “Not at all” (1) to “Very much so” (4). Higher scores indicate higher trait anxiety.

Trust in medicine was measured using two subscales used in previous research [13]. The first consisted of three items measuring trust in medicine development, e.g., “I trust the current process in which medicines are developed” (cronbach's *α* = 0.84). The second consisted of two items measuring trust in pharmaceutical companies, e.g., “I believe pharmaceutical companies act in patients' best interests” (cronbach's *α* = 0.72). Items were rated on 5‐point scale from “Strongly disagree” (1) to “Strongly Agree” (5). Higher scores indicate greater trust in medicine development and pharmaceutical companies.

#### 2.3.2 Primary Outcome: Side‐Effect Attribution

Participants completed the Side‐Effect Attribution Scale [25]. Fifty symptoms were listed and respondents selected “yes” or “no” as to whether they experienced each symptom whilst taking the oral contraceptive. For responses of “yes,” a follow‐up question asked if they believed this symptom was a side‐effect of the oral contraceptive, rated on a 5‐point Likert scale from “Definitely not a side‐effect” (1) to “Definitely a side‐effect” (5). Higher scores indicate greater attribution of side‐effects to oral contraceptive. Cronbach's alpha = 0.93.

#### 2.3.3 Secondary Outcome: Discontinuation

Discontinuation was assessed by asking respondents “Over the last 18 months have you been taking the same oral contraceptive?” with response options of Yes, No (switched to a different pill), No (switched to a different method of contraception) and No (discontinued the pill with no new method of contraception).

#### 2.3.4 Controls

Participants answered questions regarding their age, ethnicity, employment status, the type of oral contraceptive pill they had been taking in the last 18 months, and also their self‐rated health. Self‐rated health was measured using the single‐item self‐rated health scale [26] which asked respondents to rate whether their health on a 5‐point Likert scale from “Excellent” (1) to “Poor” (5).

### 2.4 Analysis

Incomplete responses were deleted after a week of inactivity in line with ethical approval that incomplete responses would be treated as withdrawn data. In initial quality checks, data from two respondents were removed for not meeting the inclusion criteria of taking an oral contraceptive within the last 18 months, and two were removed for exhibiting signs of “straightlining” in their survey responses. Predictor measures were prepared by totalling item responses to provide overall scores for each measure. Side‐effect attribution score was calculated by summing the responses to the follow‐up attribution questions and dividing the total by the number of symptoms the respondent reported as having experienced.

Linear regression analyses were conducted to investigate associations between demographic and predictor variables with side‐effect attribution score, whilst controlling for any demographic variables associated with attribution score. A binary logistic regression analysis was conducted to investigate the association between side‐effect attribution score and discontinuation (responses were coded as “1” for all discontinuation response options and “0” for continued responses to create a binary outcome variable).

## 3 Results

The final sample consisted of 275 women between 18 and 45 years old (*M* = 21.12, SD = 3.32). 89.5% of respondents were recruited from social media adverts, and 10.5% from the University Online Research Participation System. The majority of the sample was of White ethnicity (96.7%). See Table 1 for full sample characteristics. Almost all respondents (*n* = 266, 96.7%) experienced at least one symptom while taking the oral contraceptive, and their mean side‐effect attribution score was 3.46 (SD = 0.68).

**TABLE 1:** Sample characteristics and associations with side‐effect attribution score.

Variable	No (%) or mean (SD)	Side‐effect attribution score	
		B (95% CI)	*p*
Demographics			
Age	21.12 (3.32)	−0.22 (−0.50–0.07)	0.134
Ethnicity			
White	266 (96.7%)	−0.17 (−0.65–0.32)	0.499
Other ethnic group	9 (3.3%)	Reference	Reference
Employment			
Working	113 (41.1%)	0.03 (−0.14–0.20)	0.758
Not working	162 (58.9%)	Reference	Reference
Oral Contraceptive type			
Combined	176 (65.1%)	0.04 (−0.14–0.22)	0.651
Progestin‐only	96 (34.9%)	Reference	Reference
Self‐rated health status	2.30 (0.91)	0.02 (−0.08–0.11)	0.728
Psychological factors			
Belief about medicines (overuse)	11.83 (2.97)	**0.04 (0.01**–**0.06)**	0.014
Belief about medicines (harm)	8.91 (2.32)	**0.04 (0.01**–**0.08)**	0.019
Side effect expectations	3.35 (0.95)	**0.14 (0.06**–**0.23)**	0.001
Perceived sensitivity to medicines	11.13 (3.96)	**0.03 (0.01**–**0.05)**	0.013
Medicine information seeking	5.61 (2.69)	0.01 (−0.02–0.04)	0.681
Anxiety	13.92 (3.73)	0.02 (−0.001–0.05)	0.059
Trust in medicine development	10.52 (2.13)	**−0.06 (−0.09**–**0.02)**	0.004
Trust in pharmaceutical companies	5.22 (1.65)	−0.03 (−0.08–0.02)	0.243

*Note:* Bold, significant at *p* < 0.05.

The relationship between nocebo‐related factors and oral contraceptive side‐effect attribution was tested using regression analyses. As no demographic variables were significantly associated with attribution score, in text we report the unadjusted analyses. In the Supporting Information, we report the adjusted analyses, controlling for all demographic variables to see if together they accounted for any of the associations found between our psychological factors and side‐effect attribution score. No differences in the significance of the outcomes between the unadjusted and adjusted analyses were found. We found the majority of the nocebo‐related factors were associated with side‐effect attribution, specifically increased side‐effect expectations (B = 0.14, *p* < 0.01), stronger beliefs that medicines cause harm (B = 0.04, *p* < 0.05), and are overused (B = 0.04 *p* < 0.05), increased perceived sensitivity to medicines (B = 0.03, *p* < 0.05) and decreased trust in medicine development (B = −0.06, *p* < 0.01), with side‐effect expectations the strongest of these associations (see Table 1 for full results).

Over the 18‐month period, 149 (54.2%) continued and 126 (45.8%) discontinued taking their oral contraceptive. Of those who discontinued, 42 (33.3%) switched to a different oral contraceptive, 48 (38.1%) changed to a new method of contraception, and 36 (28.6%) abandoned contraceptive use entirely. Side‐effect attribution score was significantly associated with oral contraceptive discontinuation. For each one‐unit increase in side‐effect attribution score, individuals had 2.43 times higher odds (OR 1.60 to 3.68) of discontinuation.

## 4 Discussion

To our knowledge, this is the first preliminary study to investigate the link between nocebo‐related factors and oral contraceptive side effects. In this cross‐sectional study, we found that side‐effect expectations, perceived sensitivity to medicines, beliefs that medicines cause harm and are overused, and decreased trust in medicine development were associated with increased attribution of symptoms as oral contraceptive side−effects. These findings largely support the previous nocebo literature [7, 8, 14] and demonstrate a potential role that nocebo‐related factors may have in impacting oral contraceptive side‐effect experience.

The findings also demonstrated that higher side‐effect attribution scores were associated with discontinued oral contraceptive use. This highlights the potential importance of targeting these nocebo‐related factors to reduce the experiences of non‐specific side effects which are attributed to oral contraceptive use. Importantly, these factors are amenable to psychological interventions which could be employed to reduce oral contraceptive side‐effect experience and, as a result, unnecessary discontinuation. For example, positively framing side‐effect information to patients, addressing negative medication beliefs and expectations, as well as educating people about the nocebo effect has shown promise in reducing nocebo effects [27].

However, this study has several limitations. First, due to the use of opportunity sampling, we were unable to calculate a response rate, so it is unknown how representative our sample is of our population of interest. Second, the cross‐sectional nature of this study means the direction of associations cannot be established. Third, our sample predominantly consisted of young White adult oral contraceptive users and therefore cannot be generalized to older and more ethnically diverse populations. This limitation may be counteracted by the fact that oral contraceptive use is most prevalent between the ages of 20–24 [28], ‘however’ it is imperative for future research to carry out prospective studies to confirm the direction of the associations identified and recruit more diverse samples to enable generalizability. In addition, a more comprehensive set of baseline demographic and clinical variables could be explored which may also influence side‐effect experience, e.g., health literacy, prior oral contraceptive prescription, and brand of oral contraceptive.

## 5 Conclusion

Understanding the role that nocebo effects may play in oral contraceptive experience is at present limited. In this exploratory study we found that medicine‐related beliefs were associated with increased experience of oral contraceptive side−effects, demonstrating the potential role that nocebo‐related factors may have in impacting oral contraceptive side‐effect experience. These preliminary findings open an array of opportunities to assess such relationships using prospective designs and to recruit more diverse samples of oral contraceptive users to enable greater generalisability. This may lead to future opportunities to identify areas for intervention to reduce oral contraceptive side‐effect experience and, as a result, unnecessary discontinuation.

## Conflicts of Interest

The authors declare no conflicts of interest.
